# DREAM is reduced in synovial fibroblasts of patients with chronic arthritic pain: is it a suitable target for peripheral pain management?

**DOI:** 10.1186/ar2431

**Published:** 2008-05-28

**Authors:** Nataša Reisch, Andrea Engler, André Aeschlimann, Beat R Simmen, Beat A Michel, Renate E Gay, Steffen Gay, Haiko Sprott

**Affiliations:** 1 Center of Experimental Rheumatology, Department of Rheumatology and Institute of Physical Medicine, University Hospital, CH-8091 Zurich, Gloriastrasse 25, Switzerland; 2 Center for Integrative Human Physiology, University of Zurich, CH-8057 Zurich, Winterthurerstrasse 190, Switzerland; 3 RehaClinic, CH-5330 Zurzach, Quellenstrasse, Switzerland; 4 Schulthess-Klinik CH-8008 Zurich, Lengghalde 2, Switzerland

## Abstract

**Introduction:**

The endogenous pain-relieving system depends in part on the regulation of nociceptive signals through binding of opioids to the corresponding opioid receptor. Interfering with the trans-repression effect of downstream regulatory element antagonist modulator (DREAM) on the transcription of the opioid dynorphin-encoding prodynorphin (*pdyn*) gene might enhance pain relief in the periphery.

**Methods:**

Expression levels were measured in osteoarthritis (OA) synovial fibroblast-like cells (SFLCs) (n = 8) and in peripheral blood mononuclear cells (PBMCs) from OA patients (n = 53) and healthy controls (n = 26) by real-time polymerase chain reaction. Lysed OA SFLCs were analyzed by immunoprecipitation. Translation of DREAM mRNA was inhibited by small interfering RNAs (siRNAs). Expressions of DREAM, *pdyn*, and *c-fos *mRNAs were measured at 24, 48, and 72 hours after transfection.

**Results:**

The expression of DREAM mRNA was shown in both healthy and OA SFLCs as well as PBMCs. Inhibiting transcription using siRNAs led to a marked reduction in DREAM expression after 24, 48, and 72 hours. However, no significant changes in *c-fos *and *pdyn *expression occurred. In addition, DREAM mRNA expression was significantly reduced in OA patients with chronic pain (pain intensity as measured by a visual analog scale scale of greater than 40), but no *pdyn *expression was detectable.

**Conclusion:**

To our knowledge, this is the first report showing the expression of DREAM in SFLCs and PBMCs on the mRNA level. However, DREAM protein was not detectable. Since repression of *pdyn *transcription persists after inhibiting DREAM translation, DREAM appears to play no functional role in the kappa opioid receptor system in OA SFLCs. Therefore, our data suggest that DREAM appears not to qualify as a target in peripheral pain management.

## Introduction

The majority of the population is eventually confronted with severe pain during their life. The acute painful stimulus signals harm and therefore exerts a protective effect on the organism. Frequent and repetitive stimulation leads to changes on the molecular level and manifests the condition of chronic pain. Chronic pain is a devastating and widespread problem, striking one in five adults across Europe [1]. The 'Pain in Europe' study claims that more than 40% of patients suffering from chronic pain experience their pain to restrict everyday activities and to worsen the quality of life [1]. Despite ongoing intensive efforts, the control of chronic pain has not yet been achieved [2]. Arthritic diseases cause enormous burdens in terms of pain, crippling, and disability [3]. Recently, it has been demonstrated that the use of small interfering RNAs (siRNAs) to the pain-related cation channel P2X3 can be effective in the inhibition of the neuropathic pain response in an animal model [4]. A potential target to modify nociception through siRNA therapy is downstream regulatory element antagonist modulator (DREAM) [5-7]. Carrion and colleagues [8,9] showed the binding of DREAM to DNA, which implied a role in the hierarchical machinery regulating the rat dynorphin-encoding prodynorphin *(pdyn) *gene in a Ca^2+^-dependent manner. Dynorphin interacts preferably with the kappa opioid receptor (KOR), which is part of the endogenous pain-relieving machinery [10]. Thus, a diminution of the nociceptive signal is achieved and less pain is perceived [10]. Cheng and colleagues [11] demonstrated the effects of the loss of DREAM transcriptional repression *in vivo*. Higher basal levels of *pdyn *mRNA expression were noted in the lumbar spinal cord in *dream*^-/- ^mice, which showed less sensitivity in all pain paradigms tested [11]. The DNA-binding properties of DREAM have also been shown to play a role in the regulation of genes in the thyroid gland [12,13] and in hematopoetic progenitor cells [14,15]. They have also been described to regulate melatonin production in the pineal gland and the retina [16]. The genes *c-fos *[9] and *SLC8A3 *(human Na^+^/Ca^2+ ^exchanger isoform 3) [17] are regulated in part by DREAM. The repression of transcription by DREAM bound to DNA is regulated not only by changes in intracellular concentrations of Ca^2+ ^but also through the interaction with nuclear effector proteins in cAMP signaling [18,19]. In addition, the multifunctional protein DREAM was found to interact with potassium channels [20] and presenilin, a protein thought to play a major role in Alzheimer disease [21,22]. This interaction was also demonstrated *in vivo *[23].

The following questions arise: (a) Does DREAM play a role in the regulation of *pdyn *expression in chronic pain patients? (b) Does targeted inhibition of DREAM expression in synovial fibroblast-like cells (SFLCs) enhance the endogenous level of dynophin action on KOR in the periphery?

Here, we present a study on the expression of DREAM mRNA in osteoarthritis (OA) patients and the attempt to inhibit the potential signaling of DREAM in SFLCs using siRNA. The targeted inhibition of the expression of DREAM in SFLCs might enhance the endogenous level of dynorphin acting on KOR, using siRNAs locally in the periphery. If DREAM is a suitable target in pain management, it might well be the switch to reduce chronic pain in patients suffering from OA.

## Materials and methods

### Patients and tissue preparation

Synovial tissues were obtained from patients with knee OA (n = 5 females, ages 37 to 57 years, visual analog scale [VAS] score of 0 to 66, and n = 3 males, ages 27 to 38 years, VAS score of 3 to 67) who underwent synovectomy during joint replacement surgery. Synovial tissue from a healthy subject with injuries, but without arthritis, was included as a control (Department of Orthopedic Surgery, Schulthess Clinic, Zurich, Switzerland). Blood was drawn from OA patients (n = 53) and healthy controls (n = 26; RehaClinic, Zurzach, Switzerland). The procedure was approved by the local ethical committees and all patients gave written informed consent. All OA patients fulfilled the criteria of the American College of Rheumatology for the classification of OA [24].

### Isolation and culture of synovial fibroblast-like cells

The synovial tissue was minced and digested with dispase at 37°C for 60 minutes. After washing, cells were grown in Dulbecco's modified Eagle's medium (Gibco, now part of Invitrogen Corporation, Carlsbad, CA, USA) supplemented with 10% fetal calf serum, 50 IU/mL penicillin-streptomycin, 2 mM L-glutamine, 10 mM Hepes, and 0.5 μg/mL amphotericin B (all from Invitrogen Corporation). Cell cultures were maintained in a 5% CO_2_-humidified incubator at 37°C. Cultured SFLCs were used between passages 4 and 9 for all experiments described.

### Isolation of peripheral blood mononuclear cells

Peripheral blood mononuclear cells (PBMCs) from whole blood were isolated by gradient centrifugation using Ficoll Paque™ Plus (Amersham Biosciences, now part of GE Healthcare, Little Chalfont, Buckinghamshire, UK). Blood was diluted 1:2 with phosphate-buffered saline (PBS), layered on top of the corresponding amount of Ficoll Paque, and centrifuged at 450 *g *for 30 minutes at room temperature (with brakes off). The cloudy interface representing the PBMCs was transferred and washed three times in PBS, and centrifugation steps were performed at 350 *g *at room temperature for 15 minutes and twice for 10 minutes. Cells were subjected to RNA isolation.

### RNA preparation and reverse transcription-polymerase chain reaction

Total RNA was isolated with the RNeasy Mini Kit (Qiagen, Basel, Switzerland), including treatment with RNase-free DNase I (Qiagen). To generate cDNA, total RNA was reverse-transcribed in 20 μL of 1× reverse transcription-polymerase chain reaction (RT-PCR) buffer containing 5.5 mM MgCl_2_, 500 μM of each dNTP, 2.5 μM random hexamers, 0.4 U/μL RNase inhibitor, and 1.25 U/μL MultiScribe Reverse Transcriptase (Applied Biosystems, Rotkreuz, Switzerland) at 48°C for 50 minutes. Total RNAs from normal human cerebellum and spinal cord (both BD Biosciences, Clontech, Basel, Switzerland) were used as positive controls. Non-reverse-transcribed samples were used as negative controls in subsequent real-time PCR experiments. The MMVL (Moloney murine leukemia virus) reverse transcriptase (Invitrogen AG, Basel, Switzerland) and corresponding agents were used for RT of poly A^+ ^mRNA according to standard protocols [25].

### Polymerase chain reaction and cloning of DREAM amplicon

DREAM was amplified from 2 μL of generated cDNA, using specific oligonucleotides (Microsynth, Balgach, Switzerland) (Table 1) under the following conditions: 35 cycles with an initial denaturation of 5 minutes at 95°C, 30 seconds at 95°C, 30 seconds at 53°C, and 1 minute at 72°C, with a final extension for 2 minutes at 72°C. For reamplification, 5 μL of the PCR mix was subjected to the same PCR protocol using either nested primers (Microsynth) (Table 1) or the same primer set in a lower final concentration. The amplicon was purified using the QIAexII Gel extraction kit (Qiagen), cloned using the TOPO TA cloning^® ^kit (Invitrogen AG), and sequenced (Synergene Biotech GmbH, Schlieren, Switzerland).

**Table 1:** Sequences of oligonucleotides used in polymerase chain reaction (PCR) and real-time PCR as well as for the generation of small interfering RNAs

Primers for conventional DREAM PCR		
	Forward	Reverse

DREAM	5'-CCGGCTAAGGAAGTGACAAA-3'	5'-CAAAGGCGTTGAAGAGGAAG-3'
nDREAM	5'-GAAGGAGGGTATCAAGTG-3'	5'-TAAATGAGTTTGAAGGTGTC-3'
Primers for SYBR green assay real-time PCR		
	Forward	Reverse
c-fos	5'-TAAATGAGTTTGAAGGTGTC-3'	5'-ACAGGAACCCTCTAGGGAAGA-3'
Oligonucleotides for the synthesis of siRNAs		
	Sense	Antisense
siRNA1	5'-AAGGACAGGATCCACTTGACCTATAGTGAGTCGTATTA-3'	5'AAGGTCAAGTGGATCCTGTCCTATAGTGAGTCGTATTA3'
siRNA2	5'-AAGGTGAACTTGGTCTGGGCCTATAGTGAGTCGTATTA3'	5'-AAGGCCCAGACCAAGTTCACCTATAGTGAGTCGTATTA-3'
siRNA3	5'-AAGTAGAGATTAAAGGCCCACTATAGTGAGTCGTATTA-3'	5'-AAGTGGGCCTTTAATCTCTACTATAGTGAGTCGTATTA-3'
siRNA4	5'-AAGCTCATGATGTTCTCATCCTATAGTGAGTCGTATTA-3'	5'-AAGGATGAGAACATCATGAGCTATAGTGAGTCGTATTA-3'
siRNA5	5'-AAGTGTAGCAATCTGTTCACTATAGTGAGTCGTATTA-3'	5'-AAGTGAACAGATTGCTACACTATAGTGAGTCGTATTA-3'
siRNA-GFP	5'-ATGAACTTCAGGGTCAGCTTGCTATAGTGAGTCGTATTA-3'	5'-CGGCAAGCTGACCCTGAAGTTCTATAGTGAGTCGTATTA-3'
T7	5'-TAATACGACTCACTATAG-3'	

siRNA3 (binding in the coding region of exon 6) and siRNA4 (spanning the non-coding exons 8 and 9) were used to interfere with endogenous DREAM mRNA and to analyze downstream target genes of DREAM gene regulation like *pdyn *and *c-fos*. Two different primers for DREAM are given. DREAM, downstream regulatory element antagonist modulator; GFP, green fluorescence protein; siRNA, small interfering RNA.

### Real-time polymerase chain reaction

Quantification of specific mRNA was performed by single-reporter real-time PCR using the ABI Prism 7700 Sequence Detection system (Applied Biosystems). Pre-designed gene-specific primer pairs and probes for quantification of DREAM (Hs00173310_m1) and *pdyn *(Hs00225770_m1) mRNA levels were used (TaqMan^® ^Gene Expression Assays; Applied Biosystems). The level of *c-fos *mRNA was detected using primers directed against c-fos (Microsynth) (Table 1) in an SYBR green assay. 18S rRNA and GAPDH (glyceraldehyde-3-phosphate dehydrogenase) were used as endogenous controls. Relative gene expression was calculated using the comparative threshold cycle (Ct) method according to Livak and Schmittgen [26].

### Small interfering RNA generation and transfection

Different siRNAs were designed and generated according to Donzé and Picard [27]. In brief, oligonucleotides and T7 primer (listed in Table 1) were combined in 50 μL of TE (Tris ethylenediaminetetraacetic acid or Tris EDTA) (Ambion [Europe] Ltd., now part of Applied Biosystems) and annealed by heating the samples in a heating block for 2 minutes at 95°C and allowed to cool down for 6 hours in the block. The double-stranded DNA hybrid served as a template for *in vitro *transcription using T7 RNA polymerase (Stratagene Europe, Amsterdam, The Netherlands) and was incubated at 37°C for 2 hours with corresponding buffers and 2 μL of 10 mM ATP, GTP, CTP, and UTP (all from Invitrogen AG) in a total volume of 50 μL. The remaining DNA was digested with RNase-free DNase I (Roche Diagnostics, Mannheim, Germany). Sense and antisense RNAs were mixed and allowed to anneal after denaturation at 37°C for at least 1 hour. The T7 RNA polymerase synthesized small interfering double-stranded RNA (T7 siRNA) was precipitated and resuspended in 50 μL of TE buffer.

The following kits were applied for efficient transfection of SFLCs with double-stranded siRNAs: Gene Silencer™ siRNA Transfection Reagent (Gene Therapy Systems, Inc., now part of Genlantis, San Diego, CA, USA); instructions of the manufacturer were followed and applied to 24-well and 6-well formats. The Human Dermal Fibroblast Nucleofactor™ Kit (amaxa GmbH, Cologne, Germany) was used to transfect SFLCs with 1.5 μg of siRNA in a 6-well format. As described by Donzé and Picard [27] and Caplen and colleagues [28], siRNA-green fluorescence protein (GFP) served as a negative control.

### Immunoprecipitation and Western blot

SFLCs were washed with cold PBS and lysed with 50 mM Tris-HCl, pH 7.6; 1% NP-40; 150 mM NaCl; 1 mM EDTA; 1 mM phenylmethanysulphonyl-fluoride; 1 μg/mL each aprotinin, leupeptin, and pepstatin; and 1 mM Na_3_VO_4 _and incubated at 4°C for 10 minutes. Human brain tissue derived from the occipital cortex area, which was obtained from autopsy less than 4 hours after death (Institute of Neuropathology, University Hospital, Zurich, Switzerland; approved by the local ethical committee) and stored at -80°C, served as a positive control and was treated equally. For immunoprecipitation, the supernatant, obtained after centrifugation, was mixed with 1 μg of isotype matching control antibody mouse IgGs and Protein A/G plus agarose (Santa Cruz Biotechnology, Inc., Santa Cruz, CA, USA). The pre-cleared lysate was incubated overnight with anti-DREAM antibody clone 40A5 (Upstate, Lake Placid, NY, USA) (1:3,000) and Protein A/G agarose beads at 4°C. Immunoprecipitates were collected by centrifugation. Beads then were washed with ice-cold PBS, resuspended in 2 × Laemmli buffer [25], and loaded on a reducing 12.5% polyacrylamid gel. Following SDS-PAGE, the gels were blotted on Protran^® ^nitrocellulose transfer membrane (Schleicher & Schüll GmbH, Dassel, Germany), blocked, and incubated with anti-DREAM antibodies overnight. The ECL™ Western blotting detection reagents (GE Healthcare) were used after incubation with secondary goat anti-mouse horseradish peroxidase antibody (Jackson ImmunoResearch Laboratories Europe Ltd, Suffolk, UK). DREAM human recombinant protein (Abnova, Taipei, Taiwan) was used as a control.

### Statistical analysis

All data are expressed as mean ± standard error of the mean. Comparisons of two groups were made using the Mann-Whitney *U *test for unpaired data and the Wilcoxon test for paired data. For comparison of three different patient groups, data were analyzed by one-way analysis of variance (ANOVA) followed by Tukey's honest significant difference. The Shapiro-Wilk test was used to assess the distribution of the data. The level of significance was set at a *P *value of less than 0.05. All statistics were calculated using SPSS for Windows, version 11.5 (SPSS Inc., Chicago, IL, USA).

## Results

### Detection of DREAM mRNA in synovial fibroblast-like cells and peripheral blood mononuclear cells

**Figure 1:**
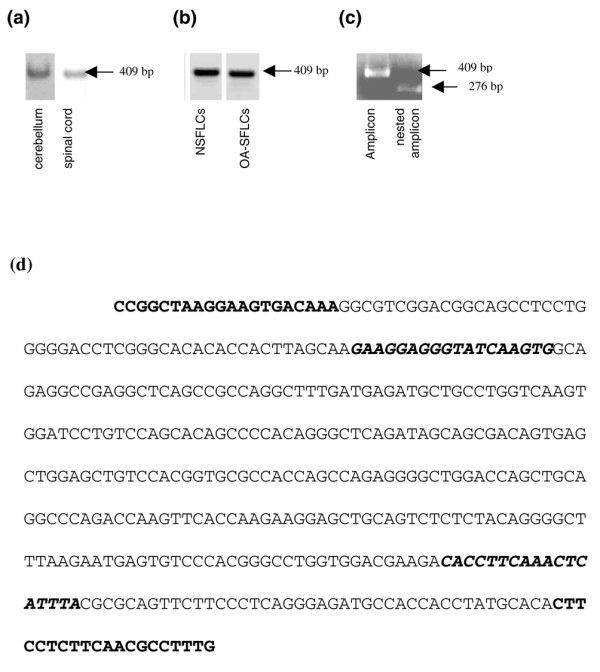
Qualitative results of reverse transcription-polymerase chain reactions (PCRs) using DREAM primer and DREAM nested primer. **(a) **DREAM amplicons of 409 base pairs (bp) in size in total RNA derived from cerebellum and spinal cord, which served as positive controls. **(b) **Amplicons of the expected size after reamplification from total RNA isolated from normal synovial fibroblast-like cells (NSFLCs) and osteoarthritis synovial fibroblast-like cells (OA-SFLCSs). **(c) **DREAM amplicon of 409 bp and the amplicon resulting from nested PCR, starting from the PCR mix, which did not show any product on the agarose gel. The size of the smaller amplicon corresponds to the expected size of 276 bp. **(d) **Sequence of the amplicon. Positions of primers are highlighted in bold (DREAM forward and reverse) and bold italics (nested DREAM forward and reverse). DREAM, downstream regulatory element antagonist modulator.

Qualitative RT-PCR with nervous system-derived RNA resulted in the amplification of a DREAM-specific transcript and served as a positive control (Figure 1a). Initial amplification of the SFLC-derived mRNA did not yield a detectable product. Reamplification, using the same settings, resulted in an amplicon that matched the positive control in size (409 base pairs [bp]) (Figure 1b). Subsequent nested PCR (amplicon size 276 bp) verified the presence of a DREAM-specific transcript in OA SFLCs and normal SFLCs (NSFLCs) (Figures 1c and 1d). All amplicons were cloned and their sequences were verified. Quantitative expression of DREAM mRNA in OA SFLCs (13.9 ± 0.6; n = 8) was measured using real-time PCR. Expression levels in neuronal tissue (13.6 ± 0.76; n = 3) and NSFLCs (13.9 ± 1.53; n = 1) served as controls. The expression of DREAM mRNA was lower in PBMCs (16.46 ± 0.16; n = 19) and synovial fluid cells, which both represent a heterogeneous pool of different cell subpopulations (data not shown).

### DREAM mRNA expression is reduced in osteoarthritis patients with high visual analog scale score

**Figure 2:**
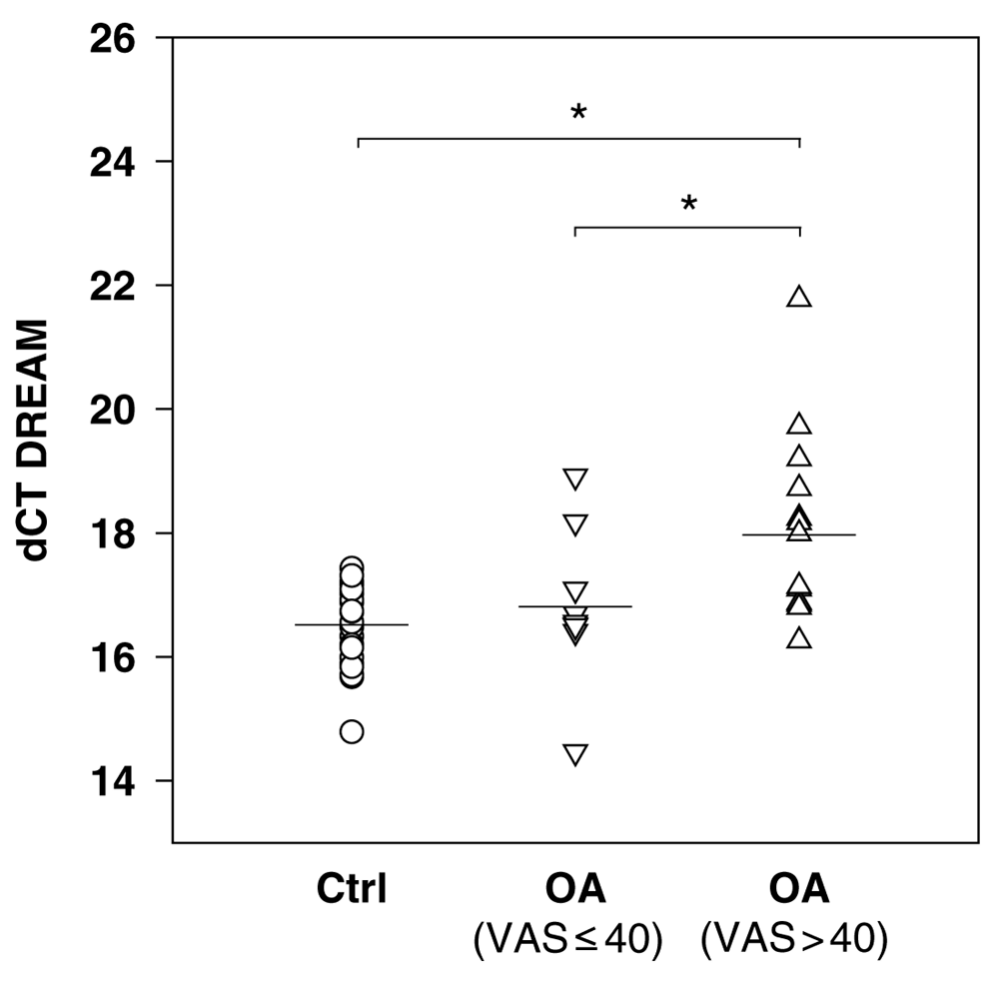
Relative DREAM gene expression in peripheral blood mononuclear cells from osteoarthritis (OA) patients and healthy controls. Relative gene expression was normalized to GAPDH (glyceraldehyde-3-phosphate dehydrogenase) and is given as delta CT (dCT) value, with higher values representing lower expression levels. DREAM gene expression was significantly lower in OA patients with a high pain score (visual analog scale [VAS] score of greater than 40; △) compared with healthy controls (○) and with OA patients with a low pain score (VAS score of less than or equal to 40; ∇). No significant differences were observed between healthy controls and OA patients with a VAS score of less than or equal to 40. Statistics: one-way analysis of variance followed by Tukey's honest significant difference (*P < 0.05). Ctrl, control; DREAM, downstream regulatory element antagonist modulator.

DREAM mRNA expression was analyzed in PBMCs from both OA patients and healthy controls. The expression of DREAM mRNA was detectable in 23/26 control subjects and in 23/53 OA patients. DREAM mRNA was significantly reduced by 63% in PBMCs from OA patients, with a pain score on the VAS (0 to 100) of greater than 40 (n = 14) compared with healthy controls. OA patients with a pain intensity of less than or equal to 40 on the VAS (n = 9) displayed no significant reduction in the expression of DREAM mRNA compared with the healthy control group (ANOVA: *F *(2,43) = 7.91; *P *< 0.001) (Figure 2). However, mRNA expression of *pdyn *was detectable neither in PBMCs derived from the healthy control group nor in PBMCs from OA patients.

### Inhibiting DREAM expression using small interfering RNAs

**Figure 3:**
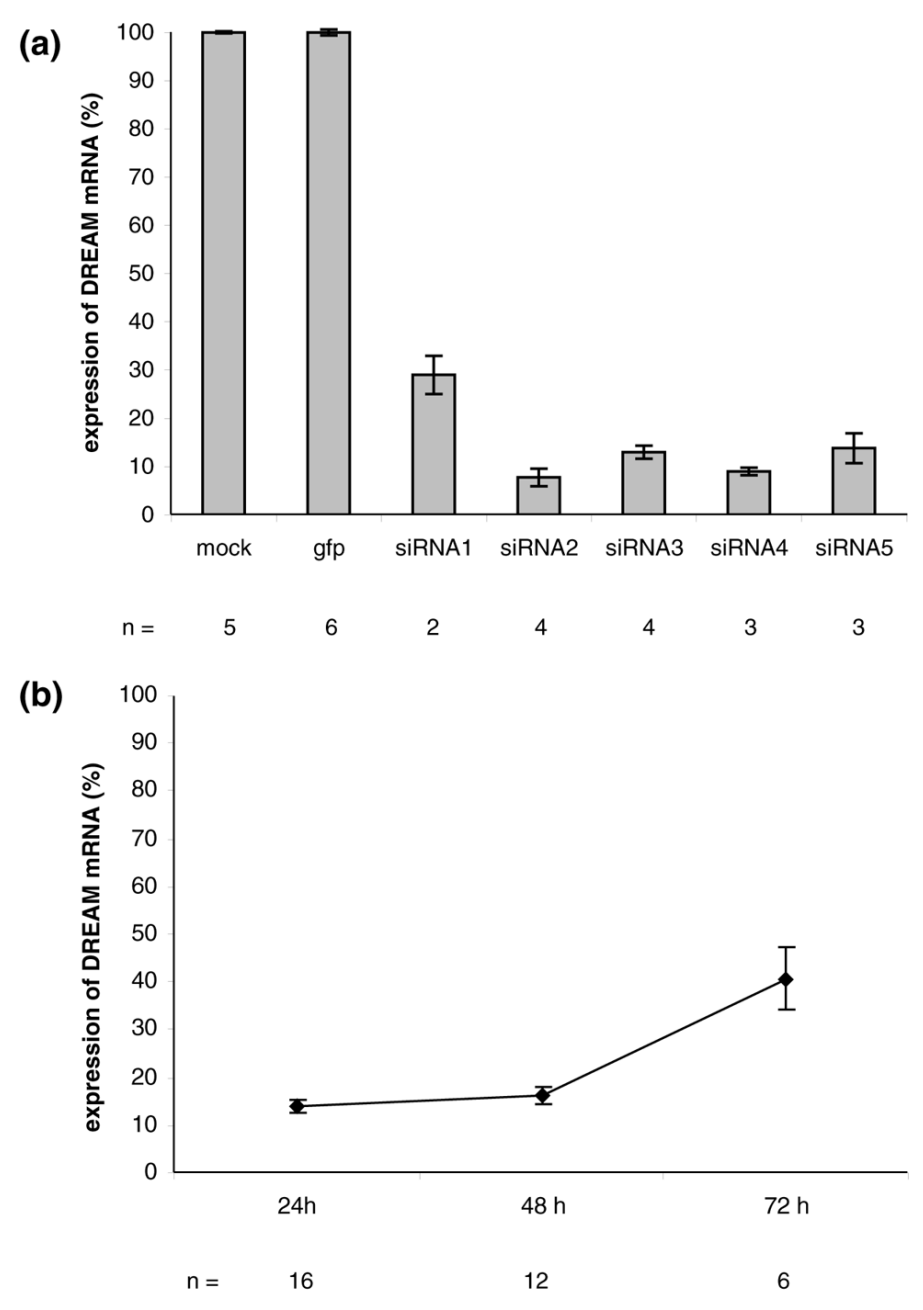
Specific downregulation of DREAM mRNA expression. **(a) **The effect of five different small interfering RNAs (siRNAs) tested. All show an overall reduction in DREAM expression of 70% to 90% compared with the expression in mock-transfected cells and cells transfected with siRNA-green fluorescence protein (100%). **(b) **Time course of reduced levels of DREAM expression in synovial fibroblast-like cells transfected with siRNA3 and siRNA4 and incubated 24, 48, and 72 hours. DREAM, downstream regulatory element antagonist modulator.

DREAM has been implicated to play a major role in pain transmission by regulating the transcription of *pdyn *in the spinal cord. DREAM^-/- ^mice showed less pain sensitivity in all paradigms tested [11]. To inhibit the blocking function of the DREAM protein on *pdyn *gene expression in SFLCs, five T7 siRNAs were designed and tested (Figure 3a). The level of DREAM expression in siRNA-GFP-transfected cells (relative expression 13.78 ± 0.67) served as baseline control and was not statistically different from mock-transfected cells (relative expression 13.65 ± 0.21; mock/siRNA-GFP *P *= 0.686) (Figure 2). DREAM mRNA was repressed to 25% ± 4% of baseline DREAM expression by siRNA1, 7.6% ± 1.8% by siRNA2, 13% ± 1.3% by siRNA3, 9% ± 0.8% by siRNA4, and 18.8% ± 3.1% by siRNA5. Although detectable DREAM transcripts were reduced to 14.17% ± 1.37% at 24 hours after transfection using siRNA3 and siRNA4 and remained at significantly low levels for an additional 24 hours (16.23% ± 1.92%), no significant changes in *pdyn *and *c-fos *expression were detected (data not shown). The level of DREAM mRNA expression was still reduced to 40.76% ± 6.74% of baseline expression at 72 hours after transfection (Figure 3b).

### Detection of DREAM protein in synovial fibroblast-like cells

**Figure 4:**
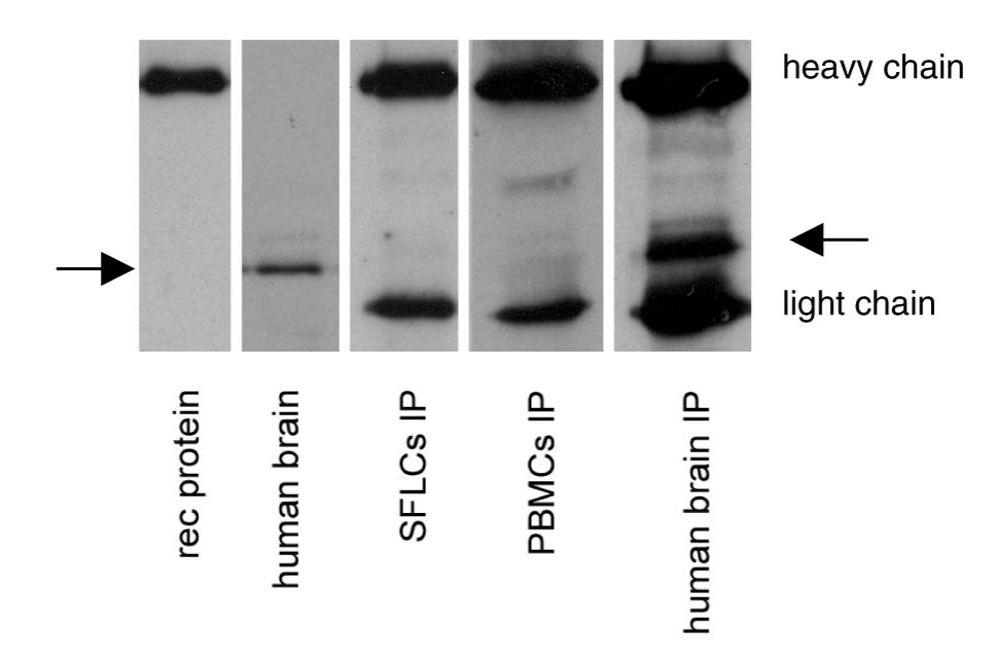
Immunoprecipitation of DREAM protein from different tissues (arrows). The DREAM antibody recognizes the glutathione S-transferase-tagged recombinant protein (10 ng; predicted size of 54 kDa) and the native protein from neuronal tissue (human occipital cortex). The immunoprecipitations show the two antibody bands detected by the secondary goat anti-mouse antibody (heavy and light chains), and in the last lane, resembling the immunoprecipitation from neuronal tissue, a DREAM-specific signal of the expected size (~30 kDa) was detectable. DREAM, downstream regulatory element antagonist modulator; IP, immunoprecipitation; PBMC, peripheral blood mononuclear cell; SFLC, synovial fibroblast-like cell.

The monoclonal mouse anti-human DREAM antibody clone 40A5 precipitated DREAM protein from human brain tissue, whereas no positive signal for DREAM protein could be detected in OA SFLCs and PBMCs (Figure 4).

## Discussion

DREAM, also known as calsenilin and KChIP3, is a member of the recoverin/neuronal calcium sensor family of nuclear calcium-binding proteins and so far has mainly been known to be expressed in the nervous system [29-31]. To our knowledge, this is the first report that demonstrates the presence of DREAM transcripts in OA SFLCs (Figure 1) as well as PBMCs and synovial fluid cells. DREAM was detected on the mRNA level on both a qualitative and a quantitative basis. *In vitro *DREAM transcription could be reduced significantly for more than 48 hours in SFLCs using siRNAs (Figure 3). However, the two target genes of DREAM transcriptional repression, *pdyn *and *c-fos*, displayed no increase in gene transcription. The basal transcription level of *pdyn *is very low in SFLCs. The expression levels of neither *pdyn *nor *c-fos *displayed significant changes, and contrary to what was expected, no increase in the level of expression was detected [11,32]. We observed minor variations in *c-fos *expression levels, which could not be attributed to the suppression of DREAM mRNA since the relative expression of *c-fos *in other non-DREAM siRNA-transfected SFLCs showed similar fluctuations. Thus, the *in vitro *knockout of DREAM might not be sufficient to ensure the transcription of *pdyn *in SFLCs compared with other models [33]. Additional factors might be necessary to initiate the transcription of both reporter genes in the analyzed cell type. Moreover, no protein was detectable with the antibodies used in this study (Figure 4). The concentration of DREAM might have been the limiting factor. The presence of DREAM in neuronal tissue could be shown in all experiments. Due to the very low endogenous level of protein, other publications dealing with DREAM *in vitro *experiments report the use of stably transfected cell lines to analyze the function and interactions of DREAM [18,19,34-37].

It has been demonstrated that immune cell-derived opioids play an important role in peripheral analgesia (reviewed in [38,39]). Leukocytes containing β-endorphin, methionine-enkephalin, and dynorphin-A migrate to the site of injury and/or inflammation where the opioid peptides are released and help to inhibit pain [40-42]. Therefore, we expected to find elevated *pdyn *mRNA levels in PBMCs derived from patients suffering from pain. But no *pdyn *mRNA was detected. In addition, contradicting the theory of DREAM action on pain relief, a reduction of the expression level of DREAM was shown in PBMCs from OA patients with a VAS score of greater than 40 (Figure 2). Less DREAM mRNA was detected in the group of patients suffering from strong and persistent pain.

*In vitro *and *in vivo *experiments show a reduction of DREAM mRNA; in both cases, no changes in levels of *pdyn *mRNA were detected. It cannot be ruled out that these negative findings were due to concentrations of transcript near the detection limit of the methods used. Nonetheless, the transcriptional inhibition of DREAM mRNA did not lead to a changed expression of the chosen reporter genes in *in vitro *experiments using siRNA. In addition, in the *in vivo *situation, a reduction of DREAM expression coincides with enhanced pain. Reduced DREAM mRNA expression appears not to be sufficient to relieve pain and/or counteract other mechanisms induced by chronic pain, which possibly include dramatic changes in the transcriptome in conditions of chronic pain. The reduction of DREAM and the sustained release of dynorphin could also be a part of an increase in pain perception, similar to the observation that opiate administration paradoxically can induce hyperalgesia [43,44].

## Conclusion

The aim to knock out DREAM as a transcriptional repressor in SFLCs in chronic pain, a major feature of OA, to induce the transcription of *pdyn *and the subsequent release of dynorphin could not be demonstrated. In addition to no significant changes in the expression level of the target gene *pdyn *in SFLCs, the presence of the *pdyn *transcript could not be detected in PBMCs. Therefore, the applied approach to increase endogenous dynorphin in the periphery appears not to be feasible, although increased expression of *pdyn *has been demonstrated in the spinal cord of *dream*^-/- ^mice [11]. However, it has to be taken into account that an ambivalent role of dynorphin has been described in the central nervous system, where higher amounts of dynorphin lead to enhanced pain [44-46]. It is nevertheless of importance that the gene product itself does not appear to play a role in the inherent KOR system previously described in SFLCs [47]. Therefore, DREAM is not a target to locally reduce the intensity of chronic pain in patients with arthritis.

## Abbreviations

ANOVA = analysis of variance; bp = base pairs; DREAM = downstream regulatory element antagonist modulator; EDTA = ethylenediaminetetraacetic acid; GFP = green fluorescence protein; KOR = kappa opioid receptor; NSFLC = normal synovial fibroblast-like cell; OA = osteoarthritis; PBMC = peripheral blood mononuclear cell; PBS = phosphate-buffered saline; PCR = polymerase chain reaction; pdyn = prodynorphin; RT-PCR = reverse transcription-polymerase chain reaction; SFLC = synovial fibroblast-like cell; siRNA = small interfering RNA; TE = Tris ethylenediaminetetraacetic acid or Tris EDTA; VAS = visual analog scale.

## Competing interests

The authors declare that they have no competing interests.

## Authors' contributions

NR and AE performed the experiments of the study and helped to write the manuscript. They contributed equally to this work. AA and REG wrote project applications to the below-mentioned foundations to get financial support. BRS performed joint surgery and provided the material for the experiments. BAM developed the study design, analyzed the data, and helped to write the manuscript. SG and HS wrote project applications to the below-mentioned foundations to get financial support, developed the study design, analyzed the data, helped to write the manuscript, and decided to submit the manuscript for publication to *Arthritis Research & Therapy*. All authors discussed the data and read and approved the final manuscript.
